# Supplementary material for: Speckle-based X-ray microtomography via preconditioned Wirtinger flow
Source: Light Sci Appl. 2026 Feb 24;15:121. doi: 10.1038/s41377-025-02118-z (PMC12932712; doi:10.1038/s41377-025-02118-z)
Supplement: Supplementary file 1 — Supplementary Information for: Speckle-based X-ray microtomography via preconditioned Wirtinger flow [file 41377_2025_2118_MOESM1_ESM.pdf]

Supplementary Information for: Speckle-based X-ray microtomography via preconditioned Wirtinger flow

KyeoReh Lee*†, Herve Hugonnet†, Jae-Hong Lim, and YongKeun Park*

*Corresponding authors. Email: lee.kyeo@gmail.com; yk.park@kaist.ac.kr

†These authors contributed equally to this work.

This PDF file includes:

Supplementary Text

Table S1

Algorithm S1 to S2

Figs. S1 to S12

Captions for Videos S1 to S6

Supplementary Text

Theory on coherent speckle generation

Consider a general speckle-based X-ray microtomography setup consisting of an X-ray source, a sample, a diffuser, and a detector (Fig. S1). We draw a straight line from the source to any point on the detector

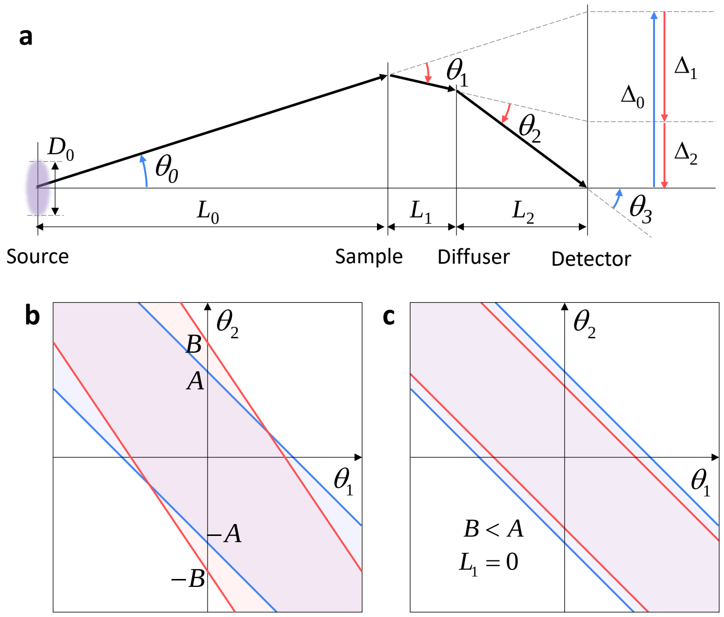

Fig. S1 Coherence speckle generation in partially coherent X-ray microtomography a, Setup diagram. The source, sample, diffuser and detector are placed from the front. The definitions of the variables used are as follows: D_0, the size of the source; θ_0, the emission angle of the source; θ_1, the diffraction angle of the sample; θ_2, the diffraction angle of the diffuser; θ_3, the detection angle at the detector; Δ_0, Δ_1, and Δ_2 are the lateral propagation distances due to θ_0, θ_1, and θ_2, respectively; and L_0, L_1, and L_2 are the distances between the elements. Note that the angles can be negative. The blue and red arrows represent positive and negative angles, respectively. **b**, Valid (θ_1, θ_2) pairs to construct a speckle grain. The blue and red lines represent the bounds of the equations S5 and S6, respectively, and the shaded area represents the (θ_1, θ_2) pairs that satisfy the equations. The constants A and B are the intercepts of θ_2, which are $\lambda/(4p) + \lambda/(2D_0)$ and $\lambda(L_0 + L_1)/(2D_0L_2) + \lambda/(2D_0)$, respectively. **c**, Same plot as **b**, but when $B < A$ and $L_1 = 0$, the overlapped area is constrained only by Eq. S6.

and describe the diffractive angles from the sample and the diffuser relative to this line, θ_1 and θ_2, respectively (Fig. S1a). The corresponding emission (θ_0) and detection (θ_3) angles are determined based on two geometric relations

$$\theta_0 + \theta_1 + \theta_2 + \theta_3 = 0 \tag{S1}$$

and

$$\Delta_0 + \Delta_1 + \Delta_2 = 0, \tag{S2}$$

where Δ_0, Δ_1, and Δ_2 are the lateral propagation distances due to θ_0, θ_1, and θ_2, respectively (Fig. S1a). Under the paraxial approximation, we have $\Delta_0 = \theta_0(L_0 + L_1 + L_2)$, $\Delta_1 = \theta_1(L_1 + L_2)$, and $\Delta_2 = \theta_2 L_2$, where L_0, L_1, and L_2 are the source-sample, sample-diffuser, and diffuser-detector distances, respectively (Fig. S1a).

To produce the coherent speckle pattern at the detector plane, the detector should not be able to distinguish our source from a coherent one. To achieve this, the emission angles should be small enough that the corresponding diffraction limit is larger than the source size (D_0)

$$\frac{\lambda}{2\,|\theta_0|} > D_0, \tag{S3}$$

where λ is the X-ray wavelength. To capture the speckle pattern without loss, the detection angle should be smaller than the Nyquist frequency of the detector ($0.5/p$), where p is the effective pixel size of the detector. Since we are measuring the intensity part of the speckle, which doubles the bandwidth, we have

$$\frac{2\,|\theta_3|}{\lambda} < \frac{1}{2p}. \tag{S4}$$

Here, we do not consider the sizes of the sample and diffuser, assuming they are much larger than $\theta_0 L_0$ and $\theta_3 L_2$, respectively. If the source is coherent enough, the size of the sample or diffuser matters, as discussed in Ref. [50].

Substituting Eqs. S3 and S4 into Eqs. S1 and S2, we can derive two conditions on θ_1, and θ_2,

$$|\theta_1 + \theta_2| < A \tag{S5}$$

and

$$\left|\left(1 + \frac{L_1}{L_2}\right)\theta_1 + \theta_2\right| < B, \tag{S6}$$

where $A = \lambda/(4p) + \lambda/(2D_0)$ and $B = \lambda(L_0 + L_1)/(2D_0 L_2) + \lambda/(2D_0)$ are the θ_2-intersects.

Two conditions are presented in Fig. S1b as blue and red shaded areas with corresponding boundaries of the same colors (solid lines). The overlapped area represents the valid (θ_1, θ_2) pairs that can construct coherent speckle on the detector. Note that if θ_1 and θ_2 have different signs, they can cancel each other and generate an acquirable coherent speckle. This is analogous to the analyzer grating in grating shearing

interferometry [13]. Since the red lines always have a steeper slope than the blue lines, the two lines always intersect, effectively reducing the overlapped area. In other words, $L_1 = 0$ is preferred to maximize the overlapped area. This is why we keep L_1 at a minimum throughout the experiments.

Since only Eq. S5 condition contains the pixel size, violating this condition would induce finer speckles than the pixel size, which is not preferred for computational processing due to aliasing. Therefore, we find it better to be constrained by Eq. S6 rather than by Eq. S5 as depicted in Fig. S1c. To achieve that condition, we need to make $B < A$ with $L_1 = 0$, which results in

$$2p\frac{L_0}{D_0} < L_2, \tag{S7}$$

which defines minimum L_2. In our experimental situation ($p = 650\,\mathrm{nm}$, $D_0 = 500\,\mu\mathrm{m}$, and $L_0 = 36\,\mathrm{m}$) the minimum L_2 is 93.6 mm, which is inconsistent with the measured speckle patterns (Fig. S2). We believe this discrepancy arises because the experimental L_0/D_0 differs from the theoretical value due to experimental perturbations and diffraction from the downstream optics, a known problem with the beamline we used.

Table S1 Speckle-based phase retrieval methods

Method	Refs.	Assumption	Min. K	Computing time[a]	Remarks
UMPA	[22, 24, 29, 30]	-	1[b]	15.2 min[c]	Slow reconstruction speed. Image resolution and quality highly depend on the window size
GF	[25]	Phase only	1	0.1 s[d]	The assumption results in no attenuation image.
MIST	[26, 28]	Single material [28]	4[b]	8.0 s[c,e]	The assumption results in coupled attenuation and phase images.
LCS	[27, 31]	-	3[b]	0.2 s[c]	Strong phase-coupling artifacts in the attenuation image.
PWF	This work	-	1	4.5 s[d]	Iterative algorithm

[a] For single precision images (1630 × 1487) using MATLAB software with a GPU (GeForce RTX 4090, NVIDIA Corp.), unless specified otherwise.

[b] $K > 10$ is used in most practical demonstrations for acceptable signal-to-noise level.

[c] $K = 12$

[d] $K = 1$

[e] The GPU is not used here since the original Python script from Ref. [28] is used as is.

Algorithm S1 Preconditioned Wirtinger flow (PWF). The vector indices r and k are used for real and reciprocal spaces, respectively. Please refer to Algorithm S2 for detailed steps in the functions used.

Input: Measured sample speckle $\{y_r\} \in \mathbb{R}^m$; diffuser transmission function $\{t_r\} \in \mathbb{C}^m$; X-ray wavelength λ; propagation lengths $L_1, L_2 \in \mathbb{R}$; intensity optical transfer function $\{\mathrm{IOTF}_k\} \in \mathbb{R}^m$; preconditioning filter $\{P_k^{-1}\} \in \mathbb{R}^m$; regularization window $\{\Gamma_k^2\} \in \mathbb{R}^m$; complex regularization parameter $\alpha \in \mathbb{C}$; step size $\eta \in \mathbb{R}$; and $m \in \mathbb{R}$ is the number of image pixels.

procedure PWF($y_r, t_r, \lambda, L_1, L_2, \mathrm{IOTF}_k, P_k^{-1}, \Gamma_k^2, \alpha, \eta$)
    $\psi_r \leftarrow 0$ ▷ Complex phase, $\psi_r = \log x_r$
    $\phi_r \leftarrow 0$
    $\mu \leftarrow 1$
    while ψ_r not converged **do**
        $\phi_r^{\mathrm{prev}} \leftarrow \phi_r$
        $\mu^{\mathrm{prev}} \leftarrow \mu$
        $f(x_r), x_r, v_r \leftarrow$ PhysicalModel($\psi_r, t_r, \lambda, L_1, L_2, \mathrm{IOTF}_k$)
        $e_r \leftarrow y_r - f(x_r)$
        $g_r \leftarrow$ WirtingerDerivative($e_r, x_r, v_r, t_r, \lambda, L_1, L_2, \mathrm{IOTF}_k$)
        $g_r \leftarrow$ Preconditioner(g_r, P_k^{-1}) + Regularizer($\psi_r, \Gamma_k^2, \alpha$)
        $\phi_r \leftarrow \psi_r - \eta g_r$
        $\mu \leftarrow \frac{1+\sqrt{1+4\mu^2}}{2}$
        $\psi_r \leftarrow \phi_r + \left(\frac{\mu^{\mathrm{prev}}-1}{\mu}\right)(\phi_r - \phi_{\mathbf{r}}^{\mathrm{prev}})$ ▷ Nesterov's accelerated gradient [51]
    end while
    return ψ_r
end procedure

Algorithm S2 Used functions in Algorithm S1.

function PhysicalModel($\psi_r, t_r, \lambda, L_1, L_2, \mathrm{IOTF}_k$)
    $x_r \leftarrow e^{\psi_r}$
    $v_r \leftarrow$ FreePropagation(x_r, λ, L_1) ▷ Sample to diffuser
    $v_r \leftarrow$ FreePropagation($t_r v_r, \lambda, L_2$) ▷ Diffuser to detector
    $f(x_r) \leftarrow$ Filter($|v_r|^2, \mathrm{IOTF}_k$) ▷ IPSF convolution
    return $f(x_r), x_r, v_r$
end function

function WirtingerDerivative($e_r, x_r, v_r, t_r, \lambda, L_1, L_2, \mathrm{IOTF}_k$)
    $g_r \leftarrow$ Filter($-e_r, \mathrm{IOTF}_k$)
    $g_r \leftarrow$ FreePropagation($v_r g_r, \lambda, -L_2$)
    $g_r \leftarrow$ FreePropagation($t_r^* g_r, \lambda, -L_1$)
    $g_r \leftarrow x_r^* g_r$
    $g_r \leftarrow g_r / \max_r |t_r|^2$
    return g_r
end function

function Preconditioner(g_r, P_k^{-1})
    $g_r'' \leftarrow \mathrm{Im}(g_r)$ ▷ Gradient for the sample phase
    $g_r'' \leftarrow$ Filter(g_r'', P_k^{-1})
    $g_r \leftarrow \mathrm{Re}(g_r) + i g_r''$
    return g_r
end function

function Regularizer($\psi_r, \Gamma_k^2, \alpha$)
    $\rho_r \leftarrow$ Filter(ψ_r, Γ_k^2)
    $\rho_r \leftarrow \mathrm{Re}(\alpha)\mathrm{Re}(\rho_r) + i\mathrm{Im}(\alpha)\mathrm{Im}(\rho_r)$
    return ρ_r
end function

function FreePropagation(x_r, λ, L)
    $w \leftarrow \sqrt{\lambda^{-2} - u^2 - v^2}$ ▷ Spatial frequency along the propagation direction
    $Q_k \leftarrow e^{i2\pi w L}$
    $x_r \leftarrow$ Filter(x_r, Q_k)
    return x_r
end function

function Filter(x_r, W_k)
    $x_r \leftarrow \mathcal{F}^{-1}\{\mathcal{F}\{x_r\} W_k\}$ ▷ $\mathcal{F}\{\cdot\}$ denotes the Fourier transform
    return x_r
end function

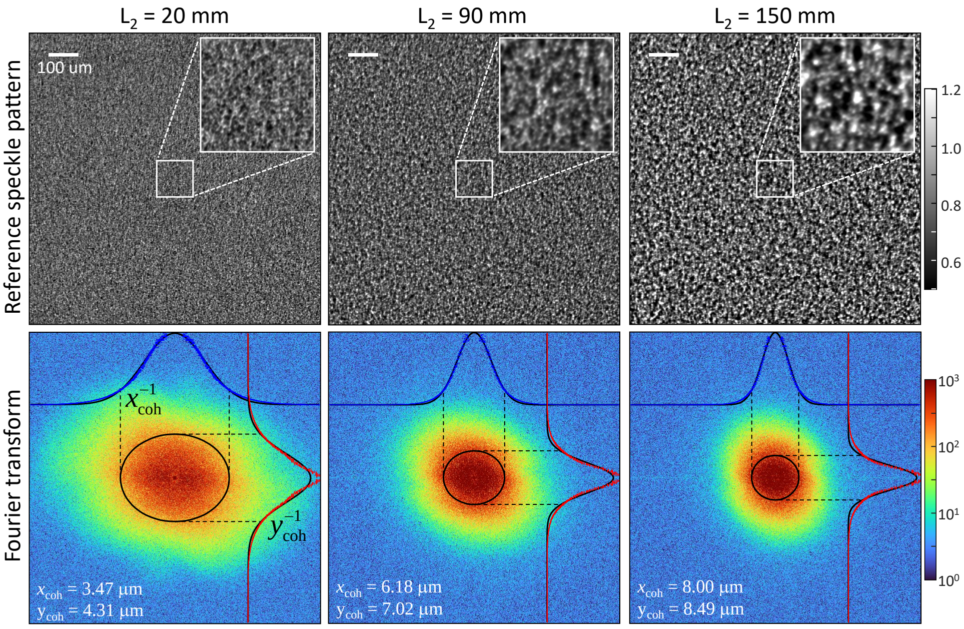

Fig. S2 Reference speckle patterns and their Fourier transforms The columns represent different L_2 values: 20 mm, 90 mm and 150 mm from left. The top row shows the raw reference speckle patterns, while the bottom row shows their Fourier transforms (i.e., power spectral density). The horizontal and vertical spatial coherence lengths (x_{coh} and y_{coh}) are given in the lower left corner, based on Eq. 5.

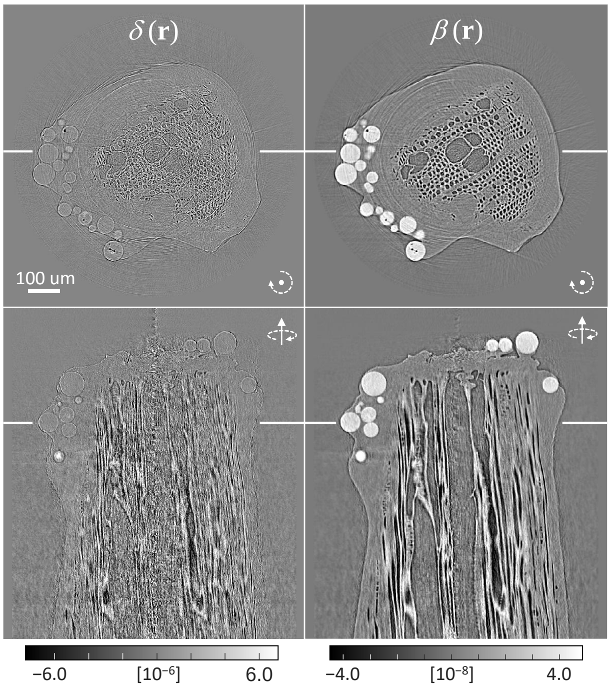

Fig. S3 Tomographic reconstruction results without the preconditioner. Without the preconditioner, the algorithm significantly underestimates the phase, resulting in completely incorrect δ and β values. All other parameters are identical to the PWF result shown in Fig. 3.

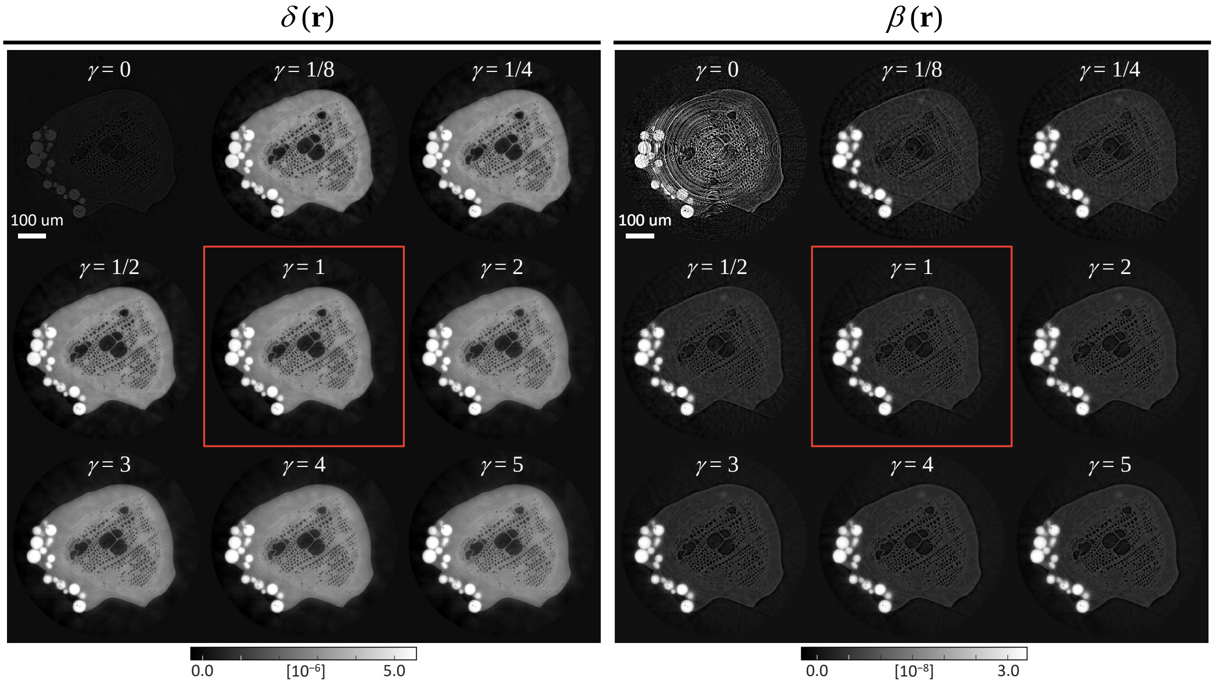

Fig. S4 Tomographic reconstruction results with different oversampling ratios (γ). Different oversampling ratios are applied to the sample transfer function (STF_k, eq. 14) used in the regularization window (Eq. 15). Subtle changes are observed except for the $\gamma = 0$ case, which means no regularization. The $\gamma = 1$ is used throughout the paper (red boxes). All other parameters are identical to the PWF result shown in Fig. 3.

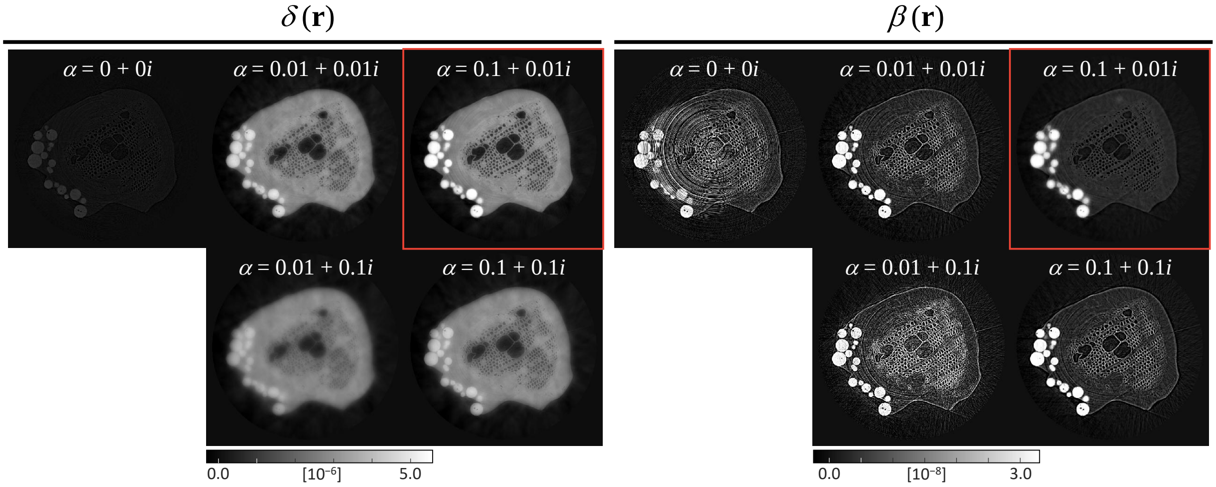

Fig. S5 Tomographic reconstruction results with different regularization parameters (α). An inappropriate regularization parameter results mainly in additional blur in $\delta(\mathbf{r})$ and edge enhancement in $\delta(\mathbf{r})$. The $\alpha = 0$ case means no regularization. The $\alpha = 0.1 + 0.01i$ is used throughout the paper (red boxes). All other parameters are identical to the PWF result shown in Fig. 3.

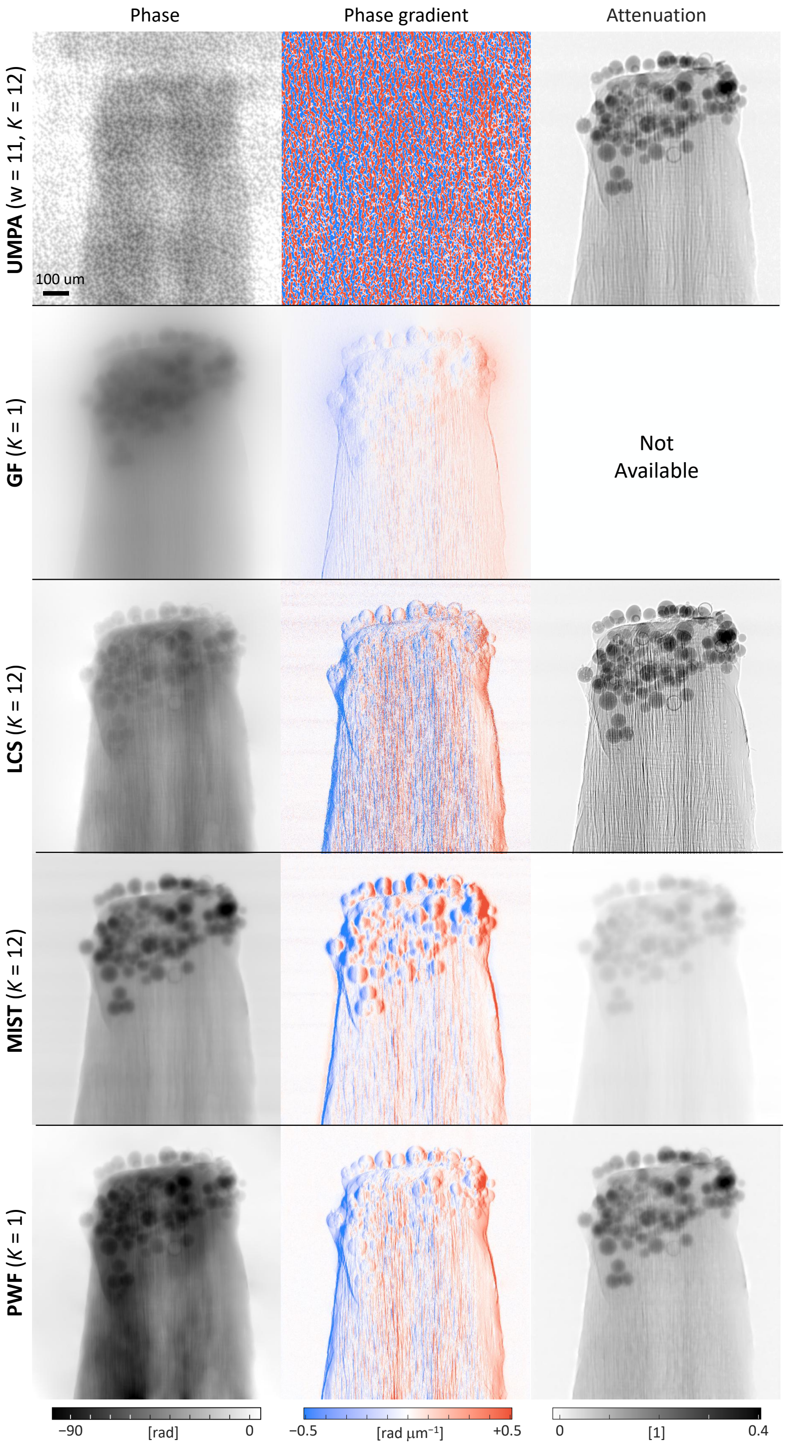

Fig. S6 Phase retrieval result from different speckle-tracking methods a, Unified modulated pattern analysis (UMPA) with $K = 12$ in different window sizes $w = 11$ [24]. **b**, geometric-flow (GF) speckle tracking with $K = 1$ [25]. An attenuation image is not available because GF neglects sample attenuation. **c**, Multimodal intrinsic speckle-tracking (MIST) with $K = 12$ [28]. An attenuation image is simply proportional to the phase because MIST assumes that a single material sample has a given ratio between the real and imaginary parts of the refractive index. **d**, Low coherence system (LCS) with $K = 12$ [27]. **e**, Preconditioned Wirtinger flow (flow) with $K = 1$. The same color scales as in Fig. 2 are used for direct comparison.

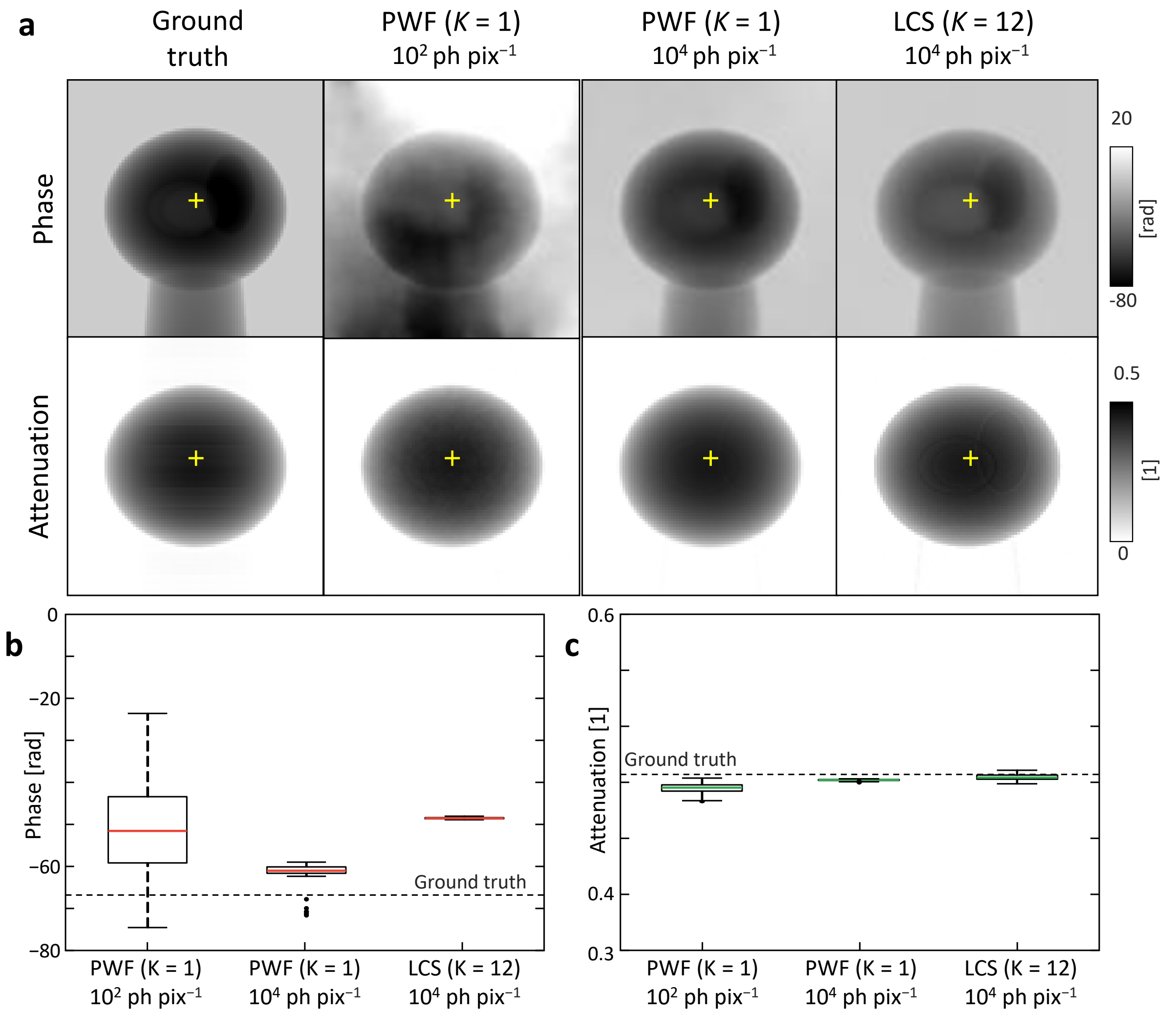

Fig. S7 Numerically simulated phase reconstruction results of preconditioned Wirtinger flow (PWF) compared with low coherence system (LCS) a, We performed numerical experiments using a phantom sample. Assuming a finite number of impinging photons per pixel, we add Poisson noise without a sample or diffuser (ph/pix, shown above). For LCS, we repeated the process 12 times with different diffuser functions. Based on these measurements, we reconstructed the phase attenuation and the images, which are shown in the top and bottom rows, respectively. **b** and **c**, The box plots show the 40 reconstructed phase (b) and attenuation (c) values at the yellow-crossed positions Fig. S7a. Each measurement used different diffuser functions while maintaining the same phantom sample.

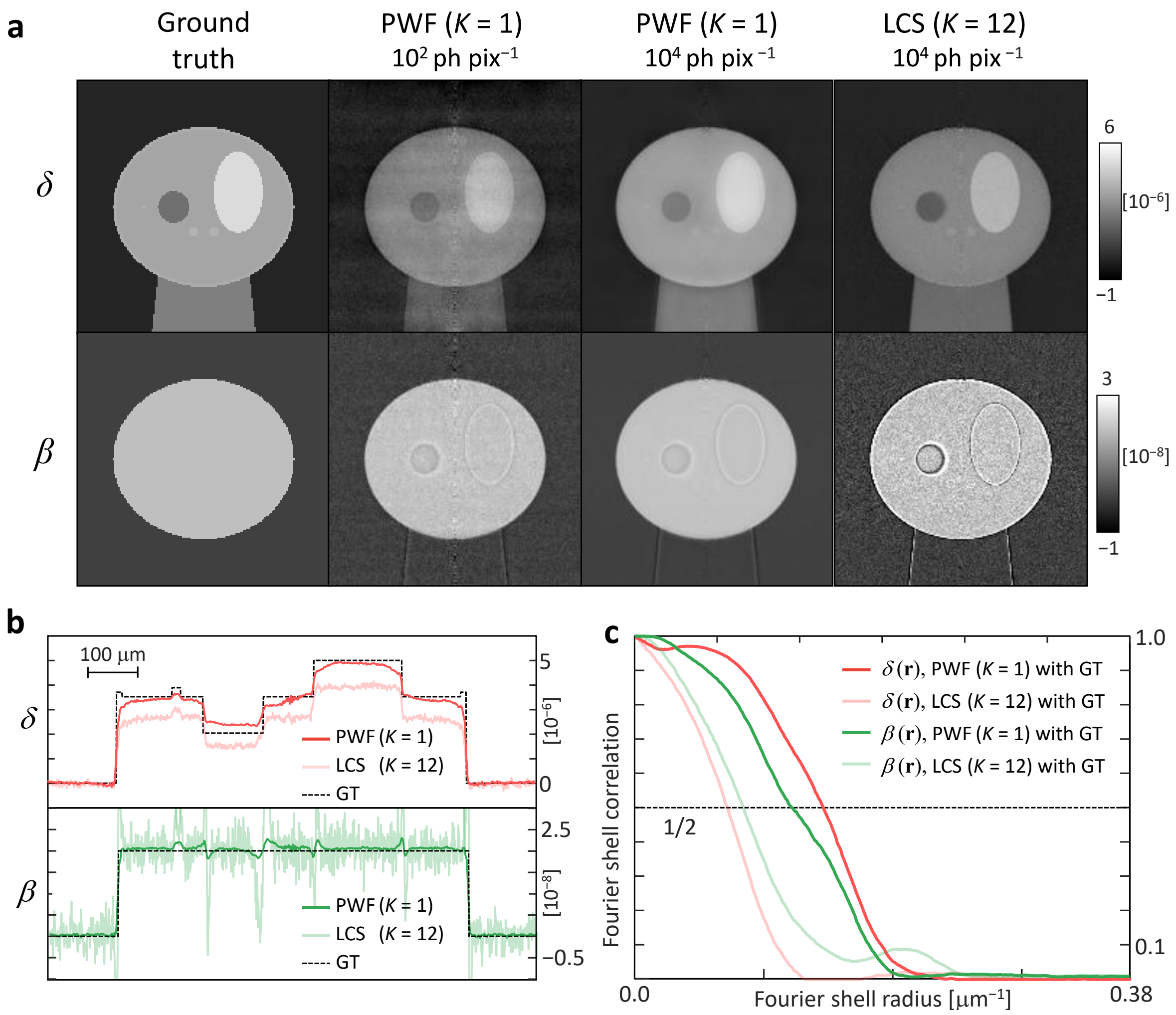

Fig. S8 Numerically simulated tomogram reconstruction results of preconditioned Wirtinger flow (PWF) compared with low coherence system (LCS) a,Building on what we did in Fig. S7, we acquire speckle images at different projection angles by numerically rotating the sample. Based on these measurements, we reconstructed the 3D refractive indices. **b**, The line profiles of the simulated results are similar to those in Fig. 3e. The PWF and LCS profiles are both from the simulated results with 10^4 ph/pix. The ground truth (GT) profile is shown as dotted lines. **c**, The Fourier shell correlation (FSC) of the simulated tomograms with the GT. Since the GT is well known, we can calculate the FSC directly with GT, which is not possible in experimental situations. The 1/2 criterion (dotted line) is shown as the resolution criterion proposed in Eq. 17.

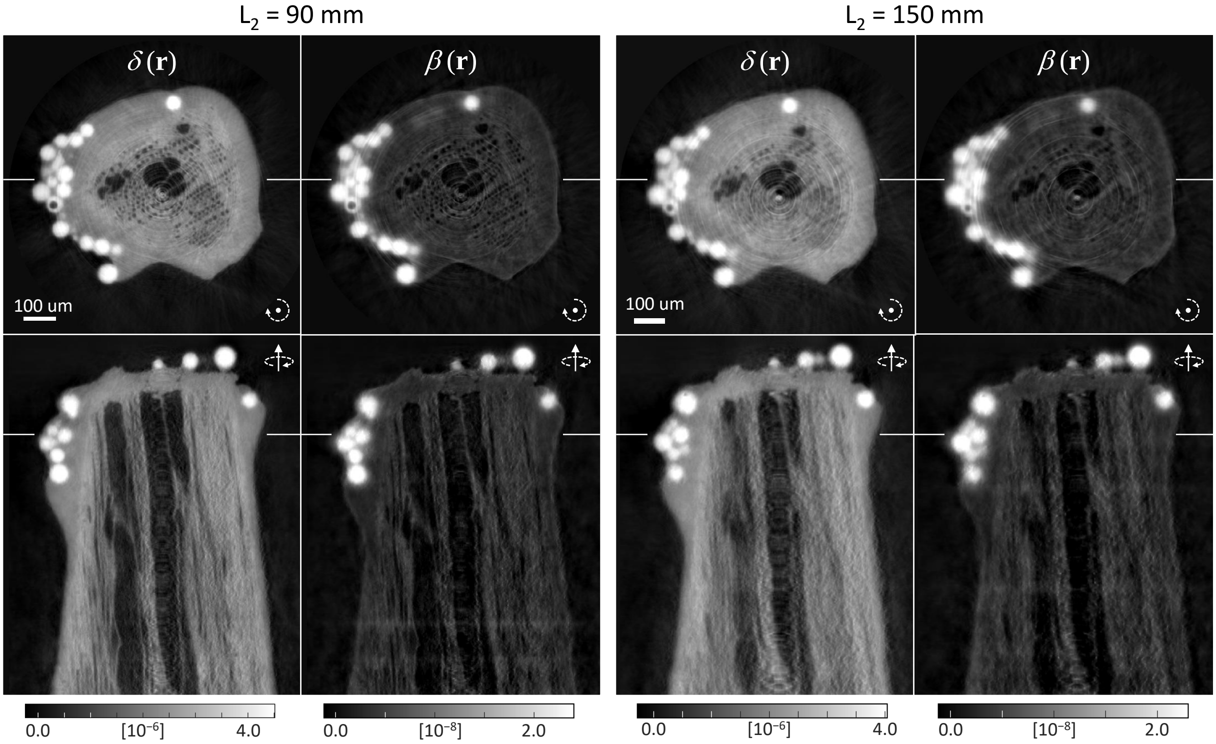

Fig. S9 Tomographic reconstruction results in different $L_2 = 90\,\mathrm{mm}$ **and** $150\,\mathrm{mm}$. Due to the increased speckle grain (Fig. S2), a decrease in spatial resolution is observed as L_2 increases.

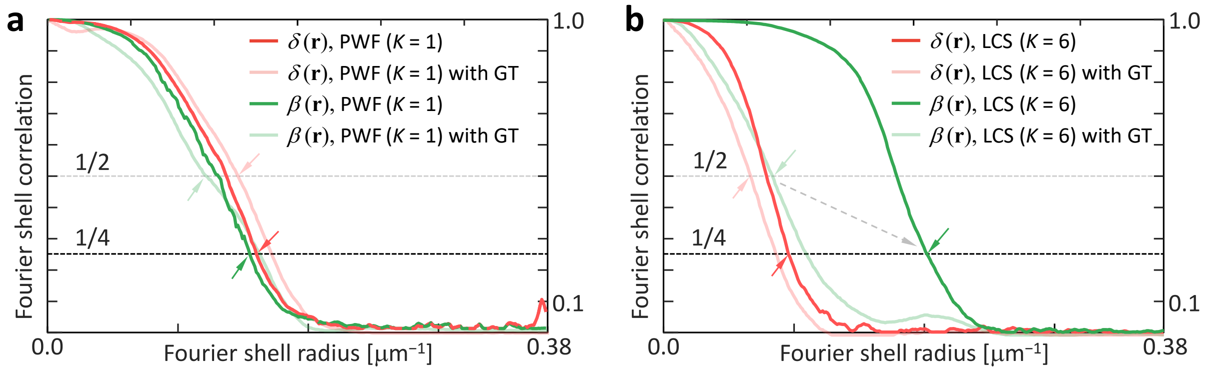

Fig. S10 Numerical simulation results of Fourier shell correlation (FSC) a and **b**, The numerical FSC results for PWF (a) and LCS (b). Two different FSCs are presented here: the FSC between independent numerical reconstructions (darker colors) and the FSC between numerical reconstructions and the ground truth (GT) (lighter colors). The former simulates the experimental FSC calculated in Fig. 3f, and the latter provides the actual resolution of the reconstructed tomogram. The resolution criteria of the two FSCs are 1/4 and 1/2, respectively (see Methods). The estimated and actual resolutions are indicated as darker and lighter arrows, respectively. A significant discrepancy in $\beta(\mathbf{r})$ of LCS is highlighted by a dotted gray arrow.

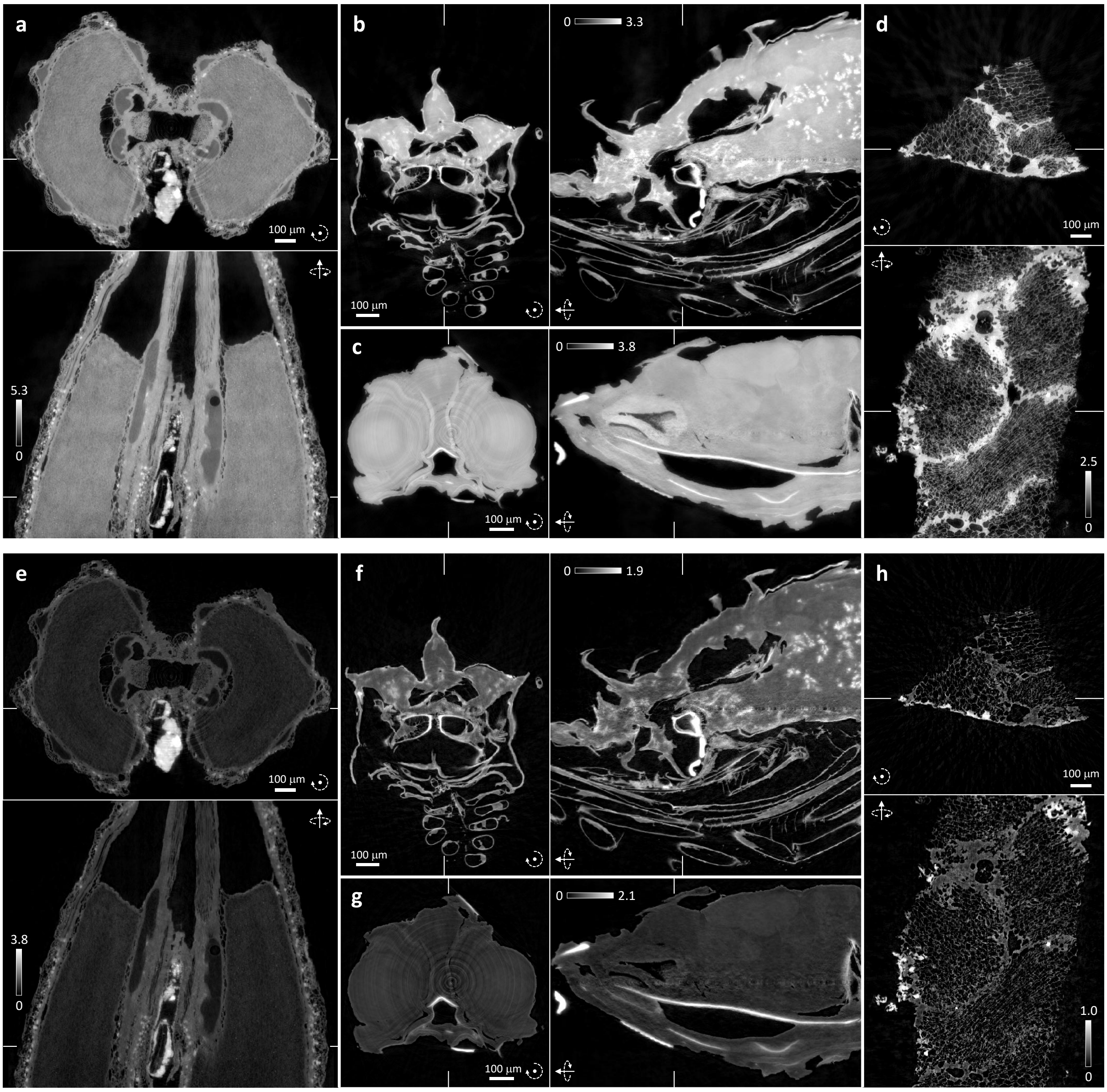

Fig. S11 The reconstructed complex refractive index—$\delta(\mathbf{r})$ and $\beta(\mathbf{r})$—of the samples in Fig. 4. **a** and **e**, A cumin seed; **b** and **f**, a dried shrimp; **c** and **g**, a dried anchovy; and **d** and **h**, a piece of cork, where **a**–**d** and **e**–**h** depicts $\delta(\mathbf{r})$ and $\beta(\mathbf{r})$, respectively. The $\delta(\mathbf{r})$ results are identical to Fig. 4, but repeated here for easier comparison. The colorbar units are 10^{-6} and 10^{-8} for **a**–**d** and **e**–**h**, respectively.

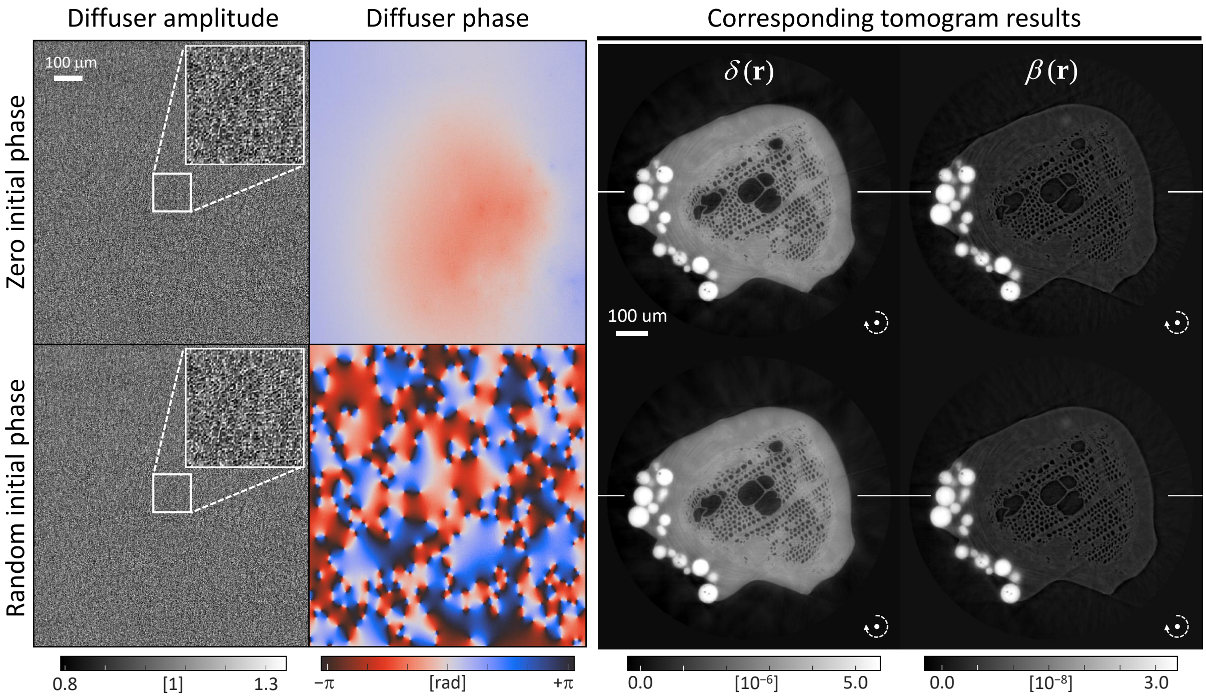

Fig. S12 Simultaneous reconstruction of the diffuser transmission function (t_r) and the corresponding tomogram results. As indicated above, the columns represent the reconstructed diffuser amplitude ($|t_r|$), reconstructed diffuser angle ($\angle t_r$), and the corresponding tomogram results. The phase of the reference speckle is initialized to zero (top row) or random (bottom row). Since all the projection angles share the same diffuser, t_r is continuously updated across all the 801 projection angles. Despite significant differences in the diffuser phase, the amplitude part remains almost identical. The diffuser phase does not significantly affect the sample field retrieval, resulting in tomogram results that are almost identical to those in Fig. 3, where the phase of the reference speckle was set to zero throughout the field retrieval sequence. All parameters are identical to the PWF result shown in Fig. 3. For the tomogram results, the same color scales as in Fig. 2 are used for direct comparison.

Video S1 Cross sections of the toothpick with glass beads. These are cross sections of the reconstructed tomogram, one perpendicular (left) and one parallel (right) to the rotation (Y-)axis. The left panel shows the cross section from top to bottom, while the right panel shows the cross section from back to front. The intersection of the two cross sections is shown as a yellow vertical line in both panels. The scale bar indicates 100 µm

Video S2 Cross sections of a cumin seed. These are cross sections of the reconstructed tomogram, one perpendicular (left) and one parallel (right) to the rotation (Y-)axis. The left panel shows the cross section from top to bottom, while the right panel shows the cross section from back to front. The intersection of the two cross sections is shown as a yellow vertical line in both panels. The scale bar indicates 100 µm

Video S3 Cross sections of a dried shrimp. These are cross sections of the reconstructed tomogram, one perpendicular (left) and one parallel (right) to the rotation (Y-)axis. The left panel shows the cross section from top to bottom, while the right panel shows the cross section from back to front. The intersection of the two cross sections is shown as a yellow vertical line in both panels. The scale bar indicates 100 µm

Video S4 Cross sections of a dried anchovy. These are cross sections of the reconstructed tomogram, one perpendicular (left) and one parallel (right) to the rotation (Y-)axis. The left panel shows the cross section from top to bottom, while the right panel shows the cross section from back to front. The intersection of the two cross sections is shown as a yellow vertical line in both panels. The scale bar indicates 100 µm

Video S5 Cross sections of a piece of cork. These are cross sections of the reconstructed tomogram, one perpendicular (left) and one parallel (right) to the rotation (Y-)axis. The left panel shows the cross section from top to bottom, while the right panel shows the cross section from back to front. The intersection of the two cross sections is shown as a yellow vertical line in both panels. The scale bar indicates 100 µm

Video S6 Experimental data measurement. Raw sample speckle measurement video performed at the 6C beamline of PLS-II in Korea.
